# Ciao1 interacts with Crumbs and Xpd to regulate organ growth in *Drosophila*

**DOI:** 10.1038/s41419-020-2564-3

**Published:** 2020-05-13

**Authors:** Jean Jung, Eunbyul Yeom, Kwang-Wook Choi

**Affiliations:** 1 Department of Biological Sciences, Korea Advanced Institute of Science and Technology (KAIST), Daejeon, 34141 Republic of Korea; 2 Metabolism and Neurophysiology Research Group, Korea Research Institute of Bioscience and Biotechnology (KRIBB), Daejeon, 34141 Republic of Korea

**Keywords:** Apoptosis, Cell growth, Cell proliferation

## Abstract

Ciao1 is a component of the cytosolic iron–sulfur cluster assembly (CIA) complex along with MMS19 and MIP18. Xeroderma pigmentosum group D (XPD), a DNA helicase involved in regulation of cell cycle and transcription, is a CIA target for iron–sulfur (Fe/S) modification. In vivo function of Ciao1 and Xpd in developing animals has been rarely studied. Here, we reveal that Ciao1 interacts with Crumbs (Crb), Galla, and Xpd to regulate organ growth in *Drosophila*. Abnormal growth of eye by overexpressing Crb intracellular domain (Crb^intra^) is suppressed by reducing the Ciao1 level. Loss of Ciao1 or Xpd causes similar impairment in organ growth. RNAi knockdown of both Ciao1 and Xpd show similar phenotypes as *Ciao1* or *Xpd RNAi* alone, suggesting their function in a pathway. Growth defects caused by *Ciao1 RNAi* are suppressed by overexpression of Xpd. Ciao1 physically interacts with Crb^intra^, Galla, and Xpd, supporting their genetic interactions. Remarkably, *Xpd RNAi* defects can also be suppressed by Ciao1 overexpression, implying a mutual regulation between the two genes. *Ciao1* mutant clones in imaginal discs show decreased levels of Cyclin E (CycE) and death-associated inhibitor of apoptosis 1 (Diap1). *Xpd* mutant clones share the similar reduction of CycE and Diap1. Consequently, knockdown of Ciao1 and Xpd by RNAi show increased apoptotic cell death. Further, CycE overexpression is sufficient to restore the growth defects from *Ciao1 RNAi* or *Xpd RNAi*. Interestingly, Diap1 overexpression in *Ciao1* mutant clones induces CycE expression, suggesting that reduced CycE in *Ciao1* mutant cells is secondary to loss of Diap1. Taken together, this study reveals new roles of Ciao1 and Xpd in cell survival and growth through regulating Diap1 level during organ development.

## Introduction

Iron–sulfur (Fe/S) clusters are essential cofactors that facilitate a number of biological processes including DNA replication and gene regulation^1^. Defects in the assembly of Fe/S clusters can result in disruptions in activities of Fe/S enzymes and iron homeostasis^1,2^. Biogenesis of Fe/S cluster is initiated in the mitochondria, and after being exported to the cytosol, they are processed by the cytosolic iron–sulfur protein assembly (CIA) machinery. In the yeast system, the WD40-repeat protein Cia1 was found as an essential member of the CIA machinery that acts in the late step of Fe/S cluster delivery to target proteins^3^.

Ciao1 was initially identified as a human protein that interacts with the zinc finger transcription factor, Wilms’ tumor suppressor protein^4^. Mutational analysis has shown that Cia1 is essential for viability in yeast and is required for maturation of cytosolic and nuclear Fe/S proteins^5^. However, in vivo function of Cia1 homologs have not been investigated in animal models. Interestingly, xeroderma pigmentosum D (XPD) is an Fe/S protein that is associated with genetic diseases such as XP, Cockayne syndrome, and tricothiodystrophy (TTD)^6–8^, implying a role of Ciao1 in Fe/S modification of XPD. XPD is a DNA helicase involved in nucleotide excision repair (NER) and transcription by forming a complex with transcription factor IIH (TFIIH). XPD has a conserved Fe/S cluster domain near the N-terminus that is essential for its proper helicase activity^9,10^. Fe/S cluster assembly of XPD by the CIA pathway is required for integration of XPD into the TFIIH complex, which allows translocation of TFIIH into the nucleus for its function in transcription and DNA repair^11,12^.

In addition to its function in NER and transcription, XPD forms a TFIIH-independent protein complex with MMS19 and MIP18 in human cells. This MMXD complex is involved in chromosome segregation by localizing to the mitotic spindle during mitosis^13^. Consistent with the role of CIA in XPD regulation, human Ciao1 was also found in the MMXD complex. Recent studies have shown that *Drosophila* Galla1 and Galla2 (MIP18 homologs) and MMS19 are required for normal chromosome segregation during nuclear division in syncytial embryo^14,15^. In this mitotic process, Galla1 and 2 show interactions with Xpd and Crumbs (Crb). The transmembrane protein Crb is required for apical basal epithelial cell polarity^16–19^ and growth regulation by affecting the Hippo signaling pathway^20–22^. Interestingly, wing overgrowth caused by Crb intracellular domain (Crb^intra^) overexpression is suppressed by reducing the level of Galla or Xpd^15^. This suggests that Crb, Galla, and Xpd are functionally related in growth regulation.

Genetic and physical interactions among Crb, Galla, and Xpd led to question whether *Drosophila* Ciao1 function is related to Crb and Galla in organ growth and whether Xpd function is regulated by Ciao1 in vivo. Here, we found that Crb^intra^ overexpression phenotype in the eye is suppressed by reducing the Ciao1 level. Also, we detected increased apoptosis in Ciao1 or Xpd reduction background. Ciao1 and Xpd are required for maintaining proper level of Cyclin E (CycE) and death-associated inhibitor of apoptosis 1 (Diap1) in imaginal discs. We demonstrate that Diap1 overexpression in *Ciao1* mutant cells induces CycE expression. This study suggests that Crb function is, in part, mediated through Ciao1-Xpd interaction for cell survival and organ growth.

## Results

### Ciao1 interacts with Crb and Galla

**Fig. 1:**
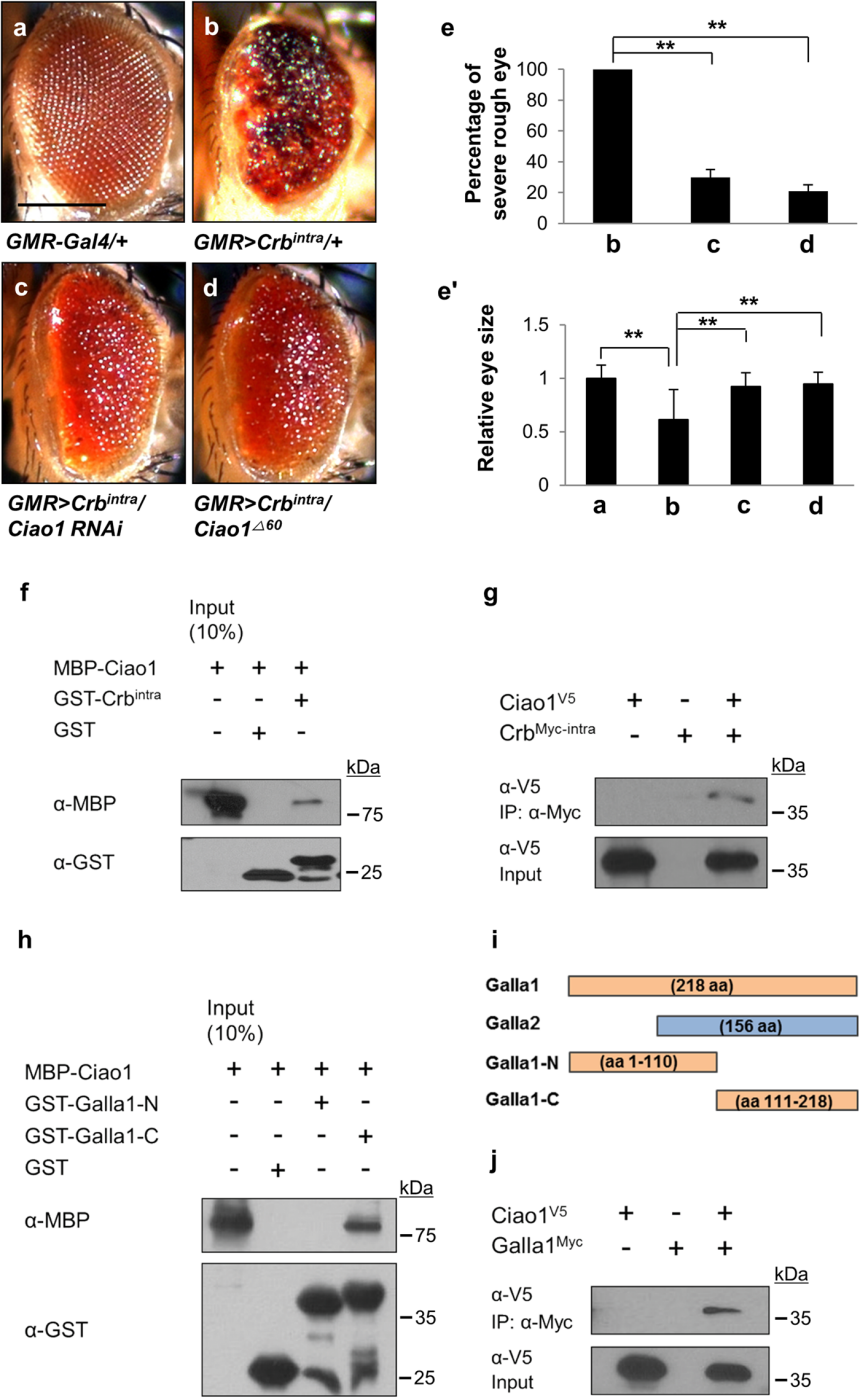
Reduced Ciao1 suppresses Crb^intra^ phenotype, and Ciao1 interacts with Crb^intra^ and Galla. **a**–**d** Genetic interaction between *crb* and *Ciao1*. **a** *GMR* > + control flies. **b** Overexpression of Crb^intra^ by *GMR-Gal4* (*GMR* > *Crb*^*intra*^) shows roughening phenotype in the eye. **c** Overexpression phenotype is partially rescued by *Ciao1 RNAi*. **d** The Crb^intra^ phenotype is also suppressed by +*/Ciao1*^*Δ60*^ mutant. Scale bar, 200 µm (**a**–**d**). **e–e′** Quantification of partial rescue of Crb^intra^ rough eye phenotype shown in **b–d**. The rescue phenotype is represented by (**e**) absence/presence of blackened ommatida on the eye surface and (**e′**) recovery in eye size. *n* ≥ 17 in each group. All data represent the mean and standard error of mean (±s.e.m.), and *p* values were calculated using the Student’s *t* test. **p < 0.01. **f** GST pull-down analysis shows direct binding between MBP-Ciao1 and GST-Crb^intra^. **g** V5-Ciao1 (Ciao1^V5^) co-immunoprecipitates with Myc-Crb^intra^ (Crb^Myc-intra^) in S2 cells. **h** MBP-Ciao1 directly binds to GST-Galla1-C and shows no binding to GST-Galla1-N. **i** Diagram of Galla1 and Galla2 full lengths and Galla1 fragments used in **h**. Galla1 has an extra N-terminal region not present in MIP18. **j** V5-Ciao1 (Ciao1^V5^) and Myc-Galla1 (Galla1^Myc^) co-immunoprecipitate in S2 cells.

**Fig. 2:**
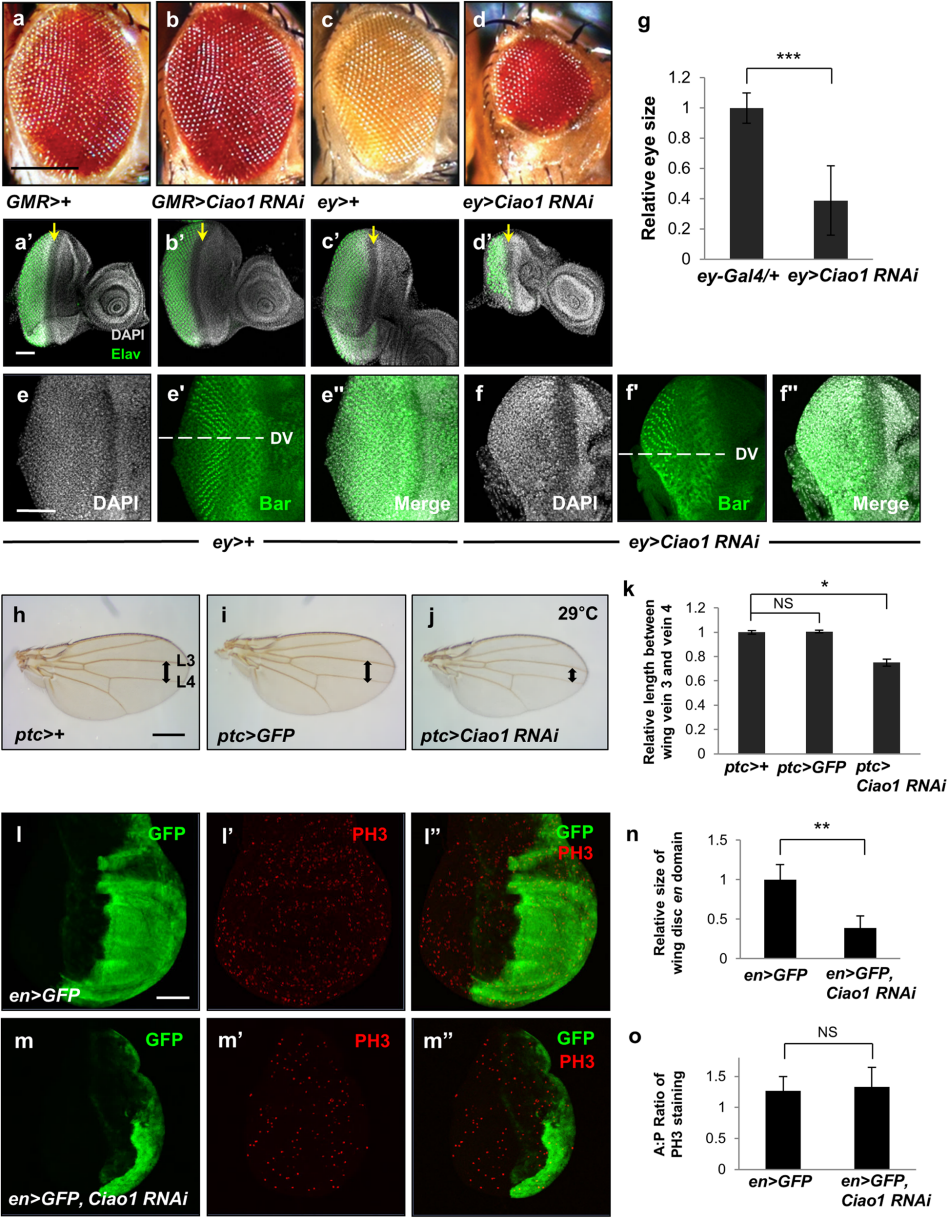
*Ciao1 RNAi* phenotypes in the eye and wing. **a**–**d** Effects of *Ciao1 RNAi* in the adult eye. **a** *GMR* > + control. **b** Knockdown of Ciao1 by *GMR-Gal4* does not affect the adult eye morphology. **c** *ey* > + control adult eye. **d** Reduction of Ciao1 in *ey* > *Ciao1 RNAi* flies display smaller and deformed eye-head structure. Scale bar, 200 µm (**a**–**d**). **a′**–**d′** Effects of *Ciao1 RNAi* in third-instar eye disc. Eye discs were stained with DAPI (gray) and anti-Elav (green). Arrows indicate position of the morphogenetic furrow. (a’) *GMR* > + eye imaginal disc as control. **b′** *GMR* > *Ciao1 RNAi* eye disc appears normal. **c′** *ey* > + control. **d′** *ey* > *Ciao1 RNAi* larva eye disc shows reduction in size with ventral loss. **e–f′** Bar staining of eye discs. The dashed line marks the dorsoventral (DV) midline. *ey* > *+* control shows normal DV pattern (**e**–**e′′**). *ey* > *Ciao1 RNAi* eye disc shows preferential loss of ventral domain below the DV boundary (**f–f′′**). Scale bar, 50 µm (**a′**–**f′′**). **g** Quantitative data shows *ey* > *Ciao1 RNAi* eyes reduced to about 40% of its normal size. *n* = 10. **h–l** Effects of *Ciao1 RNAi* in the wing at 29 °C. (h) *ptc* > *+* control wing. **i** *ptc* > *GFP* has no effect in the wing. **j** Ciao1 knockdown by *ptc-Gal4* shows reduction between longitudinal vein 3 (L3) and vein 4 (L4) region of the wing. Arrows in **h**–**j** indicate the width between L3 and L4. Scale bar, 100 µm (**h–j**). **k** Quantification of the length measured between wing vein 3 and vein 4. *n* ≥ 12 in each group. **l**–**o** Effects of *en* > *Ciao1 RNAi* on PH3 staining and the size of *en* domain. *en* > *GFP* control wing disc **l** GFP. **l′** PH3. **l′′** Merge. **m** *en* > *GFP, Ciao1 RNAi* wing discs show significant decrease in GFP expressing *en* region. **m′** PH3. **m′′** Merge. Scale bar, 50 µm (**l**–**m′′**). **n** Quantification of the GFP expressing region of *en* > *GFP, Ciao1 RNAi* wing discs. **o** The ratio of relative PH3 staining in the anterior–posterior region of the wing discs are quantified between *en* > *GFP* control and *en* > *GFP, Ciao1 RNAi* wing discs. Relative PH3 staining in the anterior and posterior domains is the number of PH3 puncta in the anterior (or posterior) divided by anterior (or posterior) area, respectively. *n* ≥ 12 in each group. All data represent the mean and standard error of mean (±s.e.m.), and *p* values were calculated using the Student’s *t* test. NS not significant (*p* > 0.05). *p < 0.05. **p < 0.01. ***p < 0.001.

**Fig. 3:**
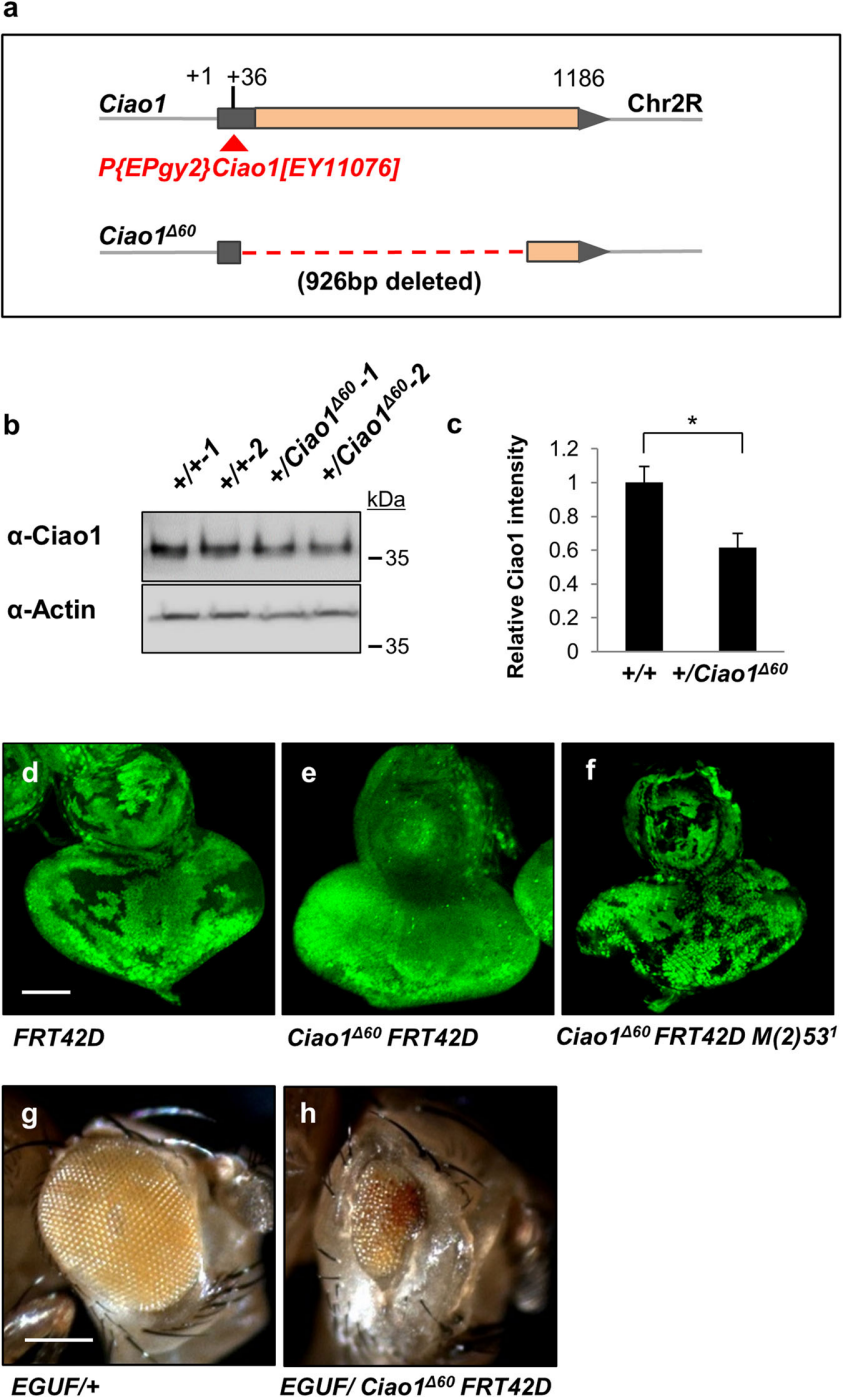
Generation and characterization of *Ciao1* deletion mutant. **a–c** Generation and verification of *Ciao1* deletion mutant. **a** Isolation of a deletion mutant *Ciao1*^*Δ60*^ by imprecise excision of P{Epgy} inserted in the 5′UTR of *Ciao1* at the 36th position from the transcription start site (+1). *The Ciao1*^*Δ60*^ mutant has a 926 bp deletion downstream of the P-element insertion site. **b** *+/Ciao1*^*Δ60*^ heterozygous adult flies (#1 and #2, each sample is extracted from two adult heterozygote flies) showed reduction in the level of Ciao1. **c** Quantification of western blot bands of **b**. *n* = 4 adult flies. All data represent the mean and standard error of mean (±s.e.m.), and *p* values were calculated using the Student’s *t* test. *p < 0.05. (**d–f**) *Ciao1* mutant clones with and without *Minute* mutation. **d** *FRT42D* control clones induced by hsFLP. **e** *Ciao1* mutant clones. Only small sized clones were produced. **f** *Ciao1* mutant clones were larger in *M*/+ background. Scale bar, 50 µm. Generation of *Ciao1* mutant clones by *EGUF*. **g** *EGUF/*+ control adult eye. **h** Adult eye phenotype of *Ciao1* mutant clones generated by *EGUF*. Scale bar, 200 µm.

Previously, we have shown that rough eye phenotype caused by overexpression of Crb^intra^ by the Gal4-UAS system^23^ is suppressed by reducing Galla1 or Galla2^15^. Since Galla proteins are homologs of mammalian MIP18 that forms a complex with Ciao1, we checked whether *Drosophila* Ciao1 is functionally related to Galla by testing its genetic interaction with Crb^intra^. Crb^intra^ overexpression by *GMR-Gal4* (*GMR* > *Crb*^*intra*^) causes severe eye roughening and reduction in eye size (Fig. 1b). Ciao1 knockdown in *GMR* > *Crb*^*intra*^ condition resulted in significant suppression of Crb^intra^ overexpression phenotype (Fig. 1c), consistent with suppression of *Crb*^*intra*^ effects by *galla1* or *galla2 RNAi*. However, *Ciao1 RNAi* by *GMR-Gal4* in the wild-type condition did not show obvious eye defects (Fig. 2b). Loss of a wild-type *Ciao1* copy by a deletion mutation (+/*Ciao1*^*Δ60*^, see Fig. 3a) also resulted in suppression of Crb^intra^ eye phenotype (Fig. 1d). The rough eye phenotype with blackened ommatidia was suppressed in ~70% of the population by *Ciao1 RNAi* (v32020 RNAi line) and 80% of the population by +/*Ciao1*^*Δ60*^ (Fig. 1e). The eye reduction by Crb^intra^ overexpression was also alleviated by *Ciao1 RNAi* (v32020) and +/*Ciao1*^*Δ60*^ (Fig. 1e′). To check the physical interaction between Ciao1 and Crb, GST pull-down and co-immunoprecipitation assays were performed. Results from GST pull-down showed direct binding between Ciao1 and Crb^intra^ (Fig. 1f). Also, V5-Ciao1 co-immunoprecipitated with Crb^Myc-intra^, suggesting these two proteins form a complex (Fig. 1g).

Information from the *Drosophila* Interactions Database suggests that Ciao1 and Galla1 and 2 interact^24^. To further reveal the interacting nature of these genes, binding experiments were carried out between Ciao1 and Galla proteins. The results showed physical interaction of Ciao1 with Galla1 but a much weaker interaction with Galla2 by GST pull-down assay (Fig. S1a). Galla1 and Galla2 are conserved, but Galla1 has an extra N-terminal region that is absent in Galla2/MIP18^15^. To identify the binding region of Ciao1, physical interaction was tested with the N-terminus (aa 1–110) and the conserved C terminus (aa 111–218)^15^ (Fig. 1i). Ciao1 directly bound with the C-terminal conserved region of Galla1 but not with the N-terminal region (Fig. 1h). In addition, co-IP assay using S2 cells showed consistent interaction between Myc-Galla1 and V5-Ciao1 (Fig. 1j).

### Ciao1 is essential for organ development

To examine the role of Ciao1 in development, we analyzed partial loss-of-function phenotypes by utilizing two independent *Ciao1 RNAi* lines (v32020 and v105939) that show similar phenotypes. We focused on *Ciao1 RNAi* effects to understand the basis for genetic interaction between *crb* and *Ciao1* in the eye. A developing eye disc in third-instar larvae consists of undifferentiated, proliferating anterior cells, and posterior cells that are mainly differentiating^25,26^. As mentioned, *Ciao1 RNAi* in differentiating cells posterior to the morphogenetic furrow did not show obvious defects on the external morphology of the adult eye (Fig. 2b). Thus, Ciao1 may not be critically required for retinal differentiation. In contrast, abnormalities were seen when utilizing *ey-Gal4* that drives Gal4 expression preferentially in undifferentiated region of the eye disc. RNAi by *ey-Gal4* (*ey* > *Ciao1 RNAi*) resulted in partial lethality during pupal stage (61% of population, *n* = 82), but all surviving flies showed strong eye reduction (Fig. 2d). The size of the *ey* > *Ciao1 RNAi* eyes were reduced to about 40% of the normal size with deformed eye-head structures (Fig. 2g). To observe developmental effects, we examined eye discs of third-instar larvae. The eye discs showed correlating phenotype to defective adult eyes where eye discs were smaller in size, often with preferential loss of the ventral region (Fig. 2d′). Eye discs were stained with the Bar antibody, a marker for R1 and R6 photoreceptor precursors, to distinguish the dorsal and ventral domains. Consistent with the shape of adult eyes, Ciao1-depleted eye discs showed more severe loss of the ventral domain (Fig. 2f–f′′). Hence, the results further support that Ciao1 is required for growth of eye discs during early larval stages.

In addition, when *Ciao1 RNAi* was crossed with *ptc-Gal4* at 29 °C, the targeted wing region along the anterior–posterior boundary between vein 3 and 4 was reduced (Fig. 2h–k). In case of *en-Gal4*, we examined wing discs prior to pupal death. Control showed GFP expression in the intact posterior compartment of wing discs. However, when the *en* domain of wing disc was knocked down by *Ciao1 RNAi*, the *GFP* expression region was dramatically reduced (Fig. 2m–n). PH3 staining was quantified, and the ratio between the anterior versus posterior region was observed. *en* > *Ciao1 RNAi* wing discs showed similar A/P ratio of PH3 signals when compared with A/P ratio of *en* > *GFP* (Fig. 2o). Taken together, the results suggest the critical role of Ciao1 in growth of different organs.

In order to test whether reduced organ size by *Ciao1 RNAi* could arise due to changes in cell size, eye discs containing *Ciao1* mutant clones were stained for the adherens junction marker, Armadillo. The results showed that cell size in *Ciao1* mutant clones did not differ compared with that of wild-type cells (Fig. S2a–b′′). In addition, adult wings of *ptc* > *Ciao1 RNAi* were observed. By taking advantage of *ptc* > *Ciao1 RNAi* phenotype, sample area was selected between L3 and L4 to measure cell density (Fig. S2c–e). The number of cells in the sampled area was compared between *ptc* > *+*, *ptc* > *GFP*, and *ptc* > *Ciao1 RNAi*. Since each wing cell produces one hair, the number of hair in a given area serves as an indicator of cell size. The results showed little difference in cell density (Fig. S2e). Thus, it appears that organ size reduction by Ciao1 loss-of-function is not due to reduced cell size.

### *Ciao1* mutant cells have a growth disadvantage

To study the effects of Ciao1 loss-of-function, we screened for *Ciao1* mutants by imprecise excision of P-element P{EPgy2}^11076^ inserted in 5′ UTR of *Ciao1*. As a result, we isolated a deletion mutant *Ciao1*^*Δ60*^ that has a 926 bp deletion downstream of the P-element insertion site (Fig. 3a). This mutant showed embryonic lethality, confirming that Ciao1 is essential for development and viability. This lethality was rescued by ubiquitous expression of Ciao1 from *UAS-Ciao1* transgene. Ciao1 antibody was generated as described in “Materials and methods”, and the produced antibody was tested for its specificity in tissues. *Ciao1*^*Δ60*^ mutant clones showed strong reduction in the Ciao1 level (Fig. S3a–a′′). Also, the level of Ciao1 was decreased in *Ciao1*^*Δ60*^*/+* heterozygous adult flies (Fig. 3b–c). *Ciao1* mutant clones induced by *heatshock* (*hs*) *Flip recombinase* (*hsFLP*)^27^ were small and often lost (Fig. 3e), suggesting that mutant cells cannot proliferate or survive well when surrounded by wild-type cells. Thus, we used the *Minute* technique^28^ to give the clone a growth advantage. By utilizing this technique, it was possible to increase the size of mutant clones comparable with that of *Ciao1*^*+*^ wild-type clones (Fig. 3f). Thus, *Ciao1* mutant cells have a growth disadvantage compared with the surrounding wild-type cells. Next, we generated *Ciao1* mutant clones by using the EGUF/hid method^29^. Most cells in the mosaic eyes generated by this method are *Ciao1* mutant cells because wild-type cells are eliminated by overexpressing the *hid* pro-apoptotic gene. Eyes containing *Ciao1 EGUF* clones were strongly reduced (Fig. 3g–h), suggesting that mutant tissues fail to grow to normal size even in the absence of competing wild-type cells. Taken together, these data suggest that Ciao1 is required for tissue growth and cell competition during development.

### *Ciao1 RNAi* phenotype is suppressed by Xpd and vice versa

**Fig. 4:**
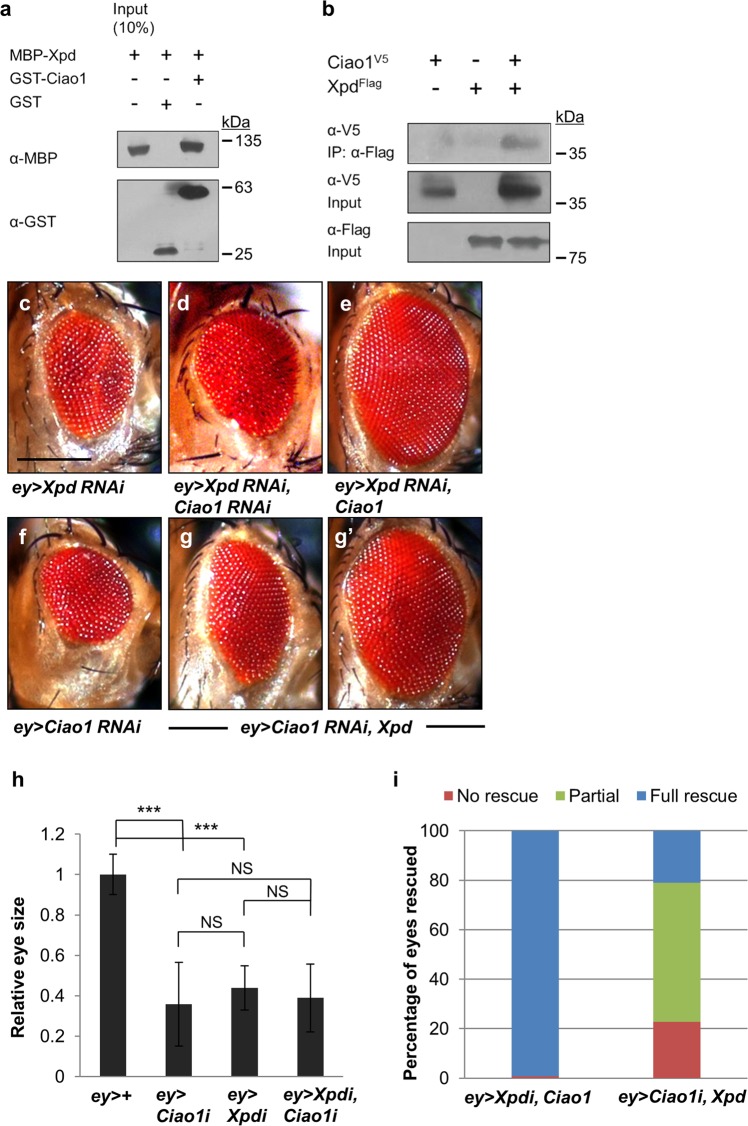
Mutual interaction between *Ciao1* and *Xpd*. **a** GST pull-down analysis shows direct binding between MBP-Xpd and GST-Ciao1. **b** Ciao1^V5^ co-immunoprecipitates with Xpd^Flag^ in S2 cells. **c–g′** Rescue effects of small eye phenotype by Ciao1 and Xpd overexpression. **c** Small eye phenotype of *ey* > *Xpd RNAi*. **d** Double depletion of Xpd and Ciao1 by RNAi shows no significant enhancement of the single RNAi eye phenotype. **e** The rescue effect of *ey* > *Xpd RNAi* by overexpression of Ciao1. **f** *ey* > *Ciao1 RNAi* flies show small eye phenotype. **g–g′** *ey* > *Ciao1 RNAi* phenotype is suppressed by Xpd overexpression but the rescue effect is varying from partial to almost full rescue. **g** Partial rescue. **g′** Almost full rescue of the small eye phenotype. Scale bar, 200 µm (**c–g′**). **h** Quantification of single or double depletion of Ciao1 and Xpd. *n* = 10. **i** Quantitative data on the rescued phenotype by overexpression of Ciao1 or Xpd. *ey* > *Xpd RNAi, Ciao1*, *n* = 48. *ey* > *Ciao1 RNAi, Xpd*, *n* = 64. *Ciao1 RNAi* and *Xpd RNAi* are abbreviated as *Ciao1i* and *Xpdi*, respectively. All data represent the mean and standard error of mean (±s.e.m.), and *p* values were calculated using the Student’s *t* test. NS not significant (*p* > 0.05). ***p < 0.001.

The Fe/S domain of human XPD is conserved in *Drosophila* Xpd, but it is unknown whether Ciao1 is functionally related to Xpd in vivo. To test their relationship, we first checked physical interaction of Ciao1 and Xpd. GST pull-down assay showed binding between Ciao1 and Xpd (Fig. 4a). Also, V5-Ciao1 co-immunoprecipitates with Flag-Xpd, suggesting they form a protein complex (Fig. 4b).

Genetic testing further revealed functional interaction between Ciao1 and Xpd. Partial loss-of-function phenotype of Xpd initially gave us clues that it could play a role in growth regulation. *Xpd RNAi* flies driven by *ey-Gal4* showed smaller eye size, and progenies driven by *nub-Gal4* or *MS1096-Gal4* showed smaller, deformed wings (Fig. S3c–e). Testing with other Gal4 lines such as *en-Gal4, ptc-Gal4*, and *ap-Gal4* all showed pupal lethality. Small eye phenotypes of *Ciao1 RNAi* and *Xpd RNAi* by *ey-Gal4* were not significantly different (Fig. 4c, f, h), although the range was larger for *ey* > *Ciao1 RNAi*. Double knockdown of Ciao1 and Xpd showed neither suppression nor enhancement of the phenotype (Fig. 4d, h), suggesting that Ciao1 and Xpd may function in the same pathway. This led us to test whether Ciao1 overexpression can rescue the eye phenotype of *Xpd RNAi* and vice versa. We observed that overexpression of Xpd can rescue *Ciao1 RNAi* eyes in 77% of the progeny, where about a third showed almost full rescue (Fig. 4g–g′). Ciao1 overexpression resulted in more efficient rescue of *Xpd RNAi* (Fig. 4e, i). Quantitative analysis shows that Ciao1 overexpression significantly rescues Xpd-depleted eyes, showing recovery to its full size (Fig. 4i).

### Loss of Ciao1 or Xpd reduces CycE

**Fig. 5:**
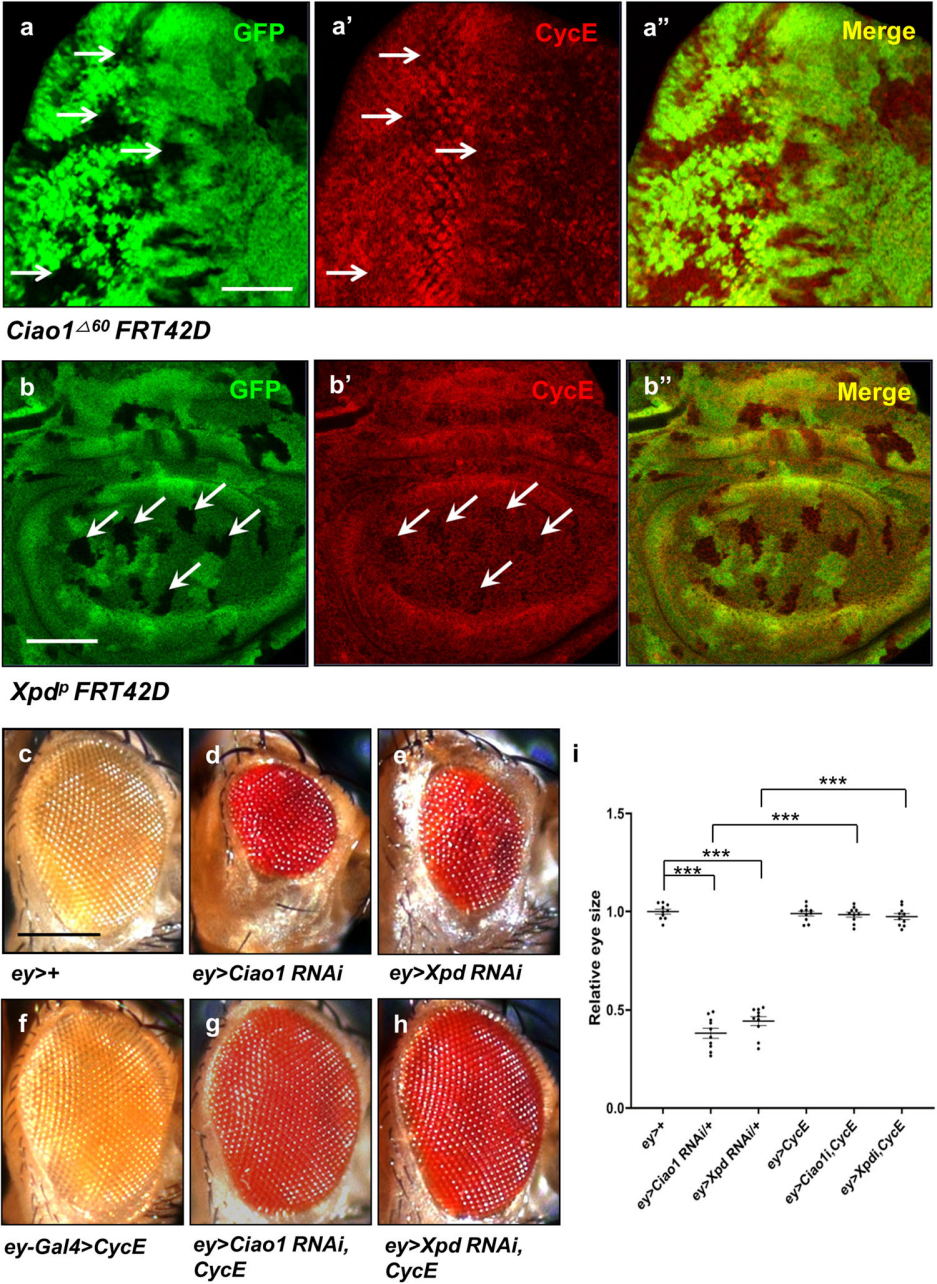
CycE reduction in *Ciao1* and *Xpd* mutant clones, and rescue effects of *Ciao1* and *Xpd RNAi* phenotype by CycE. **a–a′′** *Ciao1* mutant clones show reduced level of CycE (arrows). *Ciao1* mutant clones in the eye disc (arrows). **a** GFP. **a′** CycE. **a′′** Merge. **b–b′′** Reduced CycE staining of *Xpd* mutant clones in the wing disc (arrows). **b** GFP. **b′** CycE. **b′′** Merge. Scale bar, 50 µm (**a**–**b′′**). **c** *ey* > *+* control. **d–e** Small eye phenotype of **d** *ey* > *Ciao1 RNAi* and **e** *ey* > *Xpd RNAi*. **f** *ey* > *CycE*. **g–h** Rescue effect by CycE overexpression of **g** *ey* > *Ciao1 RNAi* and **h** *ey* > *Xpd RNAi*. **i** Quantification of the CycE rescue test. *n* = 10. *Ciao1 RNAi* and *Xpd RNAi* are abbreviated as *Ciao1i* and *Xpdi*, respectively. All data represent the mean and standard error of mean (±s.e.m.), and *p* values were calculated using the Student’s *t* test. NS not significant (*p* > 0.05). ***p < 0.001.

Reduction of organ sizes by *Ciao1 RNAi* or mutation suggests that Ciao1 is required for cell proliferation in development. Therefore, we focused on the significance of cell proliferation by checking the level of a cell cycle regulator CycE in *Ciao1* mutant cells. In eye discs, CycE is weakly expressed in all cells but its expression is enhanced in the second mitotic wave positioned along a few ommatidia columns posterior to the morphogenetic furrow^30^. Wild-type control clones did not show any changes in CycE level (Fig. S4a–a′′). In contrast, immunostaining results showed consistent reduction of CycE in most large *Ciao1* mutant clones (Fig. 5a–a′′).

Suppression of *Ciao1 RNAi* eye phenotype by Xpd overexpression and vice versa suggests that Ciao1 and Xpd are required for a related function in organ development. Based on Ciao1 results, Xpd may also be required for achieving normal CycE levels. Clonal analysis showed downregulation of CycE in most *Xpd* mutant clones (Fig. 5b–b′′). From these results, it could be inferred that Ciao1 and Xpd are required for normal CycE level to promote cell cycle progression.

### CycE rescues both *Ciao1* and *Xpd RNAi* phenotypes

Our data above suggest that both Ciao1 and Xpd are required for proper level of CycE. Thus, we tested whether reduction of CycE is primarily responsible for the growth defects caused by *Ciao1* or *Xpd RNAi*. First, we examined whether abnormal pattern of CycE in Ciao1-depleted eye discs can be recovered by overexpressing Xpd and vice versa. The eye discs of *ey* > *Xpd RNAi* or *ey* > *Ciao1 RNAi* were reduced and deformed in shape with abnormal CycE staining (Fig. S5b, d). Notably, CycE staining of the eye discs lacked its characteristic expression along the second mitotic wave. Interestingly, CycE staining pattern and size were recovered with Ciao1 overexpression in Xpd knockdown condition (Fig. S5c). Similarly, Xpd overexpression restored normal CycE pattern in Ciao1-depleted eye discs (Fig. S5e). Secondly, we tested whether CycE is sufficient to rescue the eye reduction phenotype of *Ciao1* or *Xpd RNAi*. We found that CycE overexpression fully rescued the small eye phenotype in the *Ciao1* and *Xpd RNAi* background (Fig. 5d–i).

### Loss of Ciao1 or Xpd results in apoptosis and reduced Diap1 expression

**Fig. 6:**
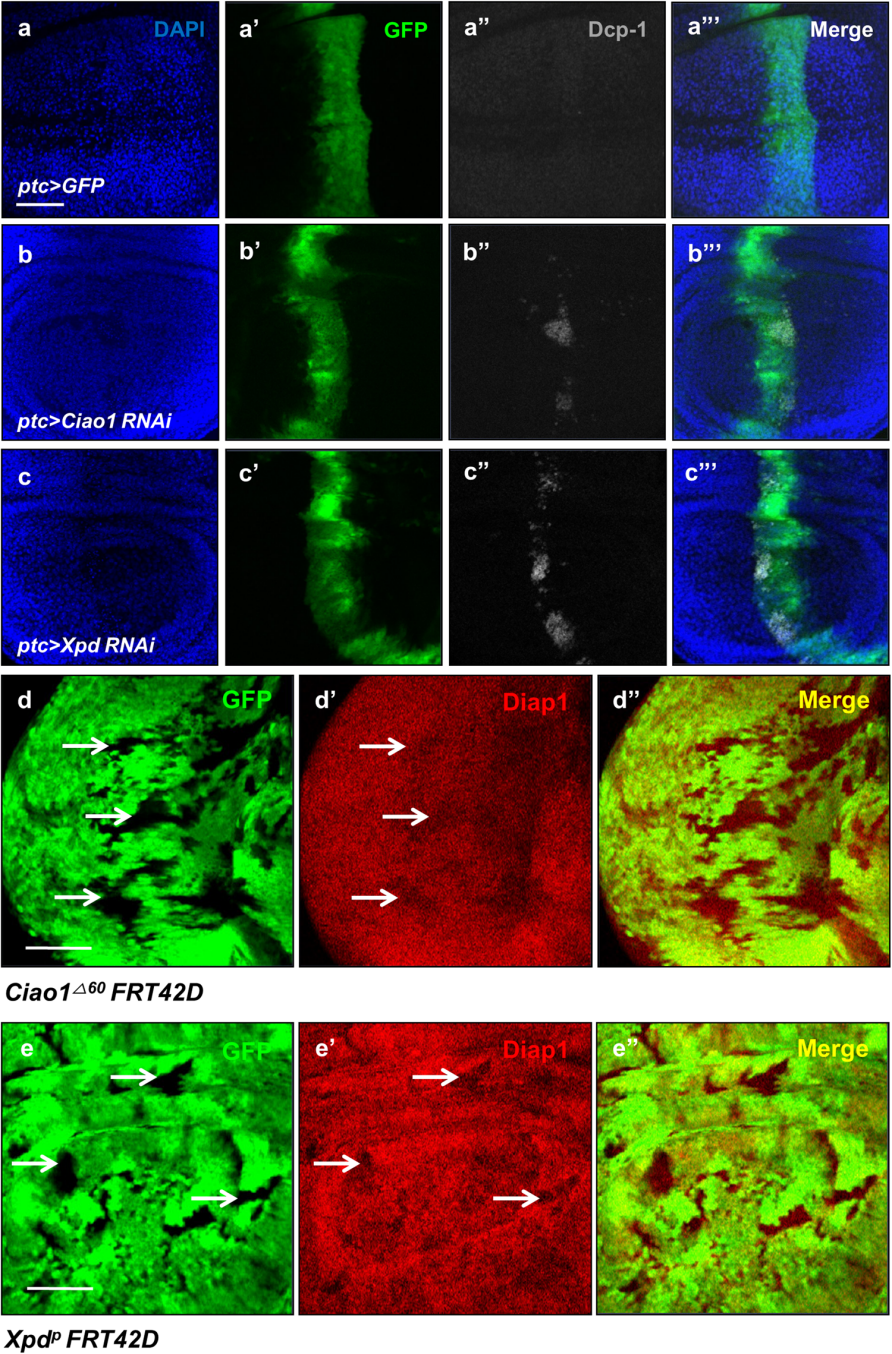
Apoptotic cell death and decreased Diap1 level in Ciao1 and Xpd reduction background. **a**–**c′′′** Apoptotic activity in *ptc* > *Ciao1 RNAi* and *ptc* > *Xpd RNAi* wing discs stained by DAPI, anti-GFP, and anti-cleaved Dcp-1 as indicated. **a**–**a′′′** Control *ptc* > *GFP* wing disc. Dcp-1 staining is undetectable. **b**–**b′′′** *ptc* > *Ciao1 RNAi*. Dcp-1 staining is increased. **c**–**c′′′** *ptc* > *Xpd RNAi* wing disc. Dcp-1 staining is increased. **d**–**d′′** Diap1 staining of *Ciao1* mutant clones. **d** GFP. **d′** Diap1 staining shows reduction (arrows). **d′′** Merge. **e**–**e′′** Diap1 staining of *Xpd* mutant clones. **e** GFP. **e′** Diap1. **e′′** Merge. Scale bar, 50 µm (**a–e′′**).

Organ size depends on cell survival as well as proliferation. To directly test whether organ reduction is a consequence of cell loss, we observed changes in caspase activity by staining discs with anti-Cleaved Dcp-1 antibody. The control wing discs did not show detectable Dcp-1 staining (Fig. 6a–a′′′). In contrast, Ciao1 knockdown in *ptc* > *Ciao1 RNAi* showed increased apoptotic activity in the A/P boundary region in ~64% of the population (Fig. 6b′′, *n* = 14). Such increases in apoptotic activity was also seen in Xpd knockdown background of *ptc* > *Xpd RNAi* in about 76% of the population (Fig. 6c′′, *n* = 17).

Based on the results that loss of Ciao1 and Xpd promotes apoptosis, we examined the level of Diap1, a caspase inhibitor necessary for cell survival^31,32^. Indeed, we found reduced levels of Diap1 in most large *Ciao1* mutant clones in the eye discs (Fig. 6d–d′′). In the wild-type control clones, such change was not seen (Fig. S4b–b′′). In addition, *Xpd* mutant clones in wing discs also showed reduction of Diap1 levels (Fig. 6e–e′′).

**Fig. 7:**
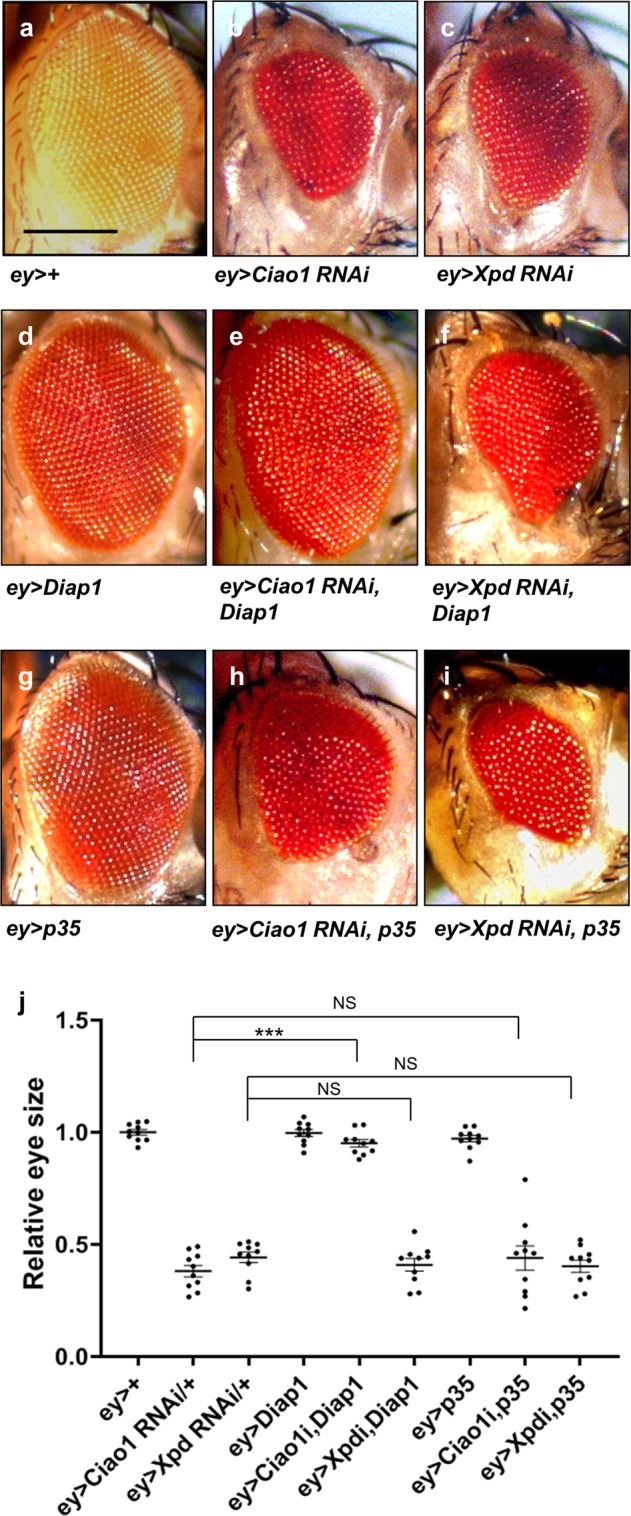
Suppression of *Ciao1* and *Xpd RNAi* by CycE, Diap1, or p35. **a** *ey* > *+* control. Small eye phenotype of **b** *ey* > *Ciao1 RNAi* and **c** *ey* > *Xpd RNAi*. **d** *ey* > *Diap1*. Rescue effect by Diap1 overexpression of **e** *ey* > *Ciao1 RNAi* and **f** *ey* > *Xpd RNAi*. **g** *ey* > *p35*. p35 overexpression has no rescue effects in **h** *ey* > *Ciao1 RNAi* and **i** *ey* > *Xpd RNAi*. Scale bar, 200 µm (**a–i**). **j** Quantification of the rescue test of all the above mentioned genotypes. *n* = 10. *Ciao1 RNAi* and *Xpd RNAi* are abbreviated as *Ciao1i* and *Xpdi*, respectively. All data represent the mean and standard error of mean (±s.e.m.), and *p* values were calculated using the Student’s *t* test. NS not significant (*p* > 0.05). ***p < 0.001.

We then tested whether inhibition of apoptosis can rescue the eye phenotype of *Ciao1* or *Xpd RNAi*. Overexpression of Diap1 or p35 apoptosis inhibitor^33^ did not affect the eye size in the wild-type background (Fig. 7d, g, j). In contrast, the small eye phenotype by *Ciao1 RNAi* could be suppressed by Diap1 overexpression (Fig. 7e, j). However, such suppression effect was not seen for *Xpd RNAi* adult eyes (Fig. 7f, j). Interestingly, overexpression of p35 could neither significantly suppress *Ciao1 RNAi* nor *Xpd RNAi* eye phenotype (Fig. 7h–j). Hence, both Ciao1 and Xpd are necessary to maintain normal Diap1 expression, and Diap1 overexpression is sufficient to rescue eye phenotypes of *Ciao1 RNAi* but not *Xpd RNAi*.

**Fig. 8:**
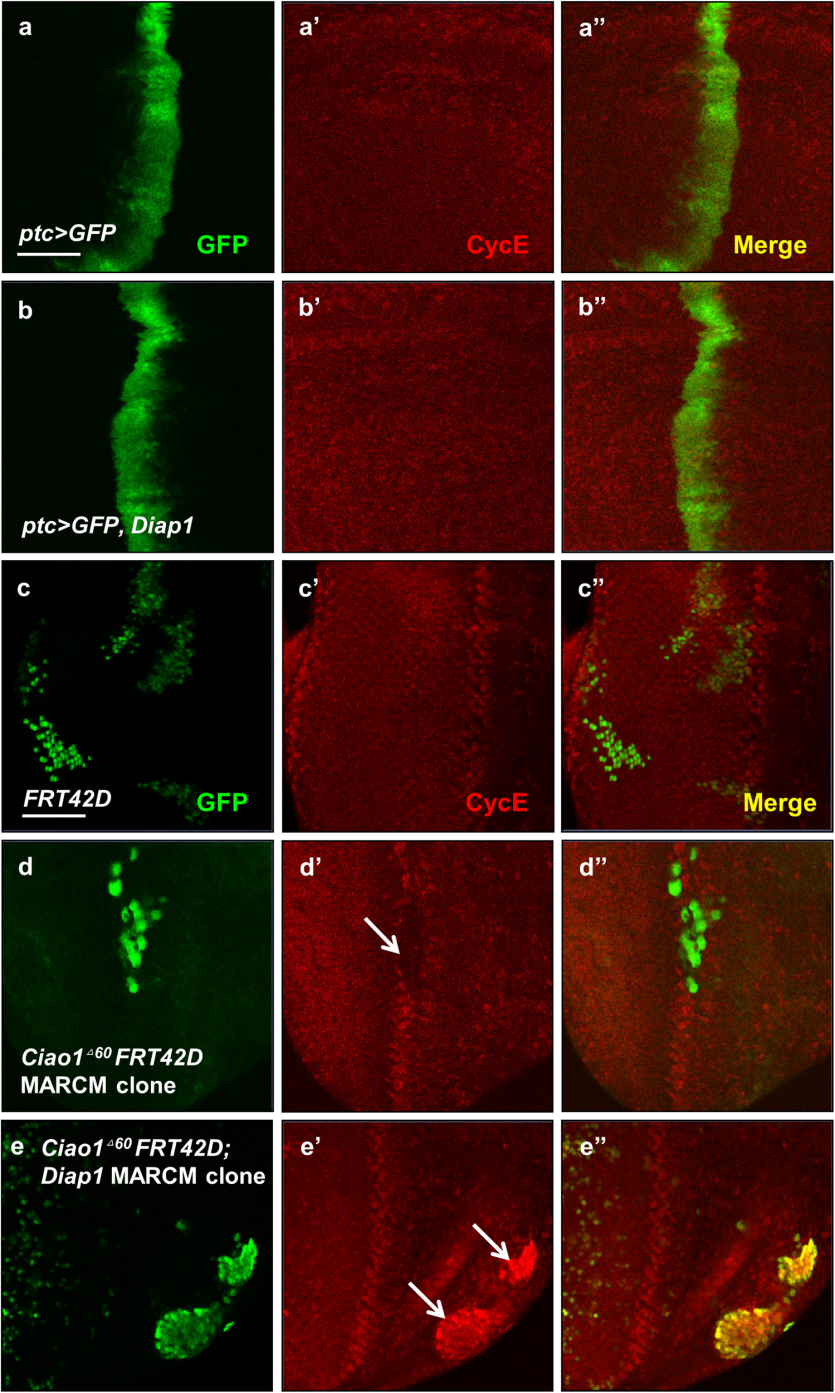
Diap1 overexpression increases CycE level in *Ciao1* MARCM clones. **a–b′′** GFP and CycE staining of control *ptc* > *GFP* and *ptc* > *Diap1*, as indicated. **a–a′′** Control wing disc. **b–b′′** *ptc* > *Diap1*. Diap1 overexpression has no changes in CycE level. **c** Wild-type control clones marked by GFP. **d–d′′** MARCM clones of *Ciao1* mutant. CycE staining is reduced in the mutant clones (arrow in **d′**). **e–e′′** MARCM clones of *Ciao1* mutants with expression of Diap1. CycE staining is elevated in the mutant clones under the expression of Diap1 (arrows in **e′**). Scale bar, 50 µm (**a–e′′**).

Since *Ciao1 RNAi* phenotype can be suppressed by Diap1 overexpression, it is possible that Diap1 overexpression may allow *Ciao1* mutant cells to restore the normal level of CycE. First, we confirmed that Diap1 overexpression has no effect on CycE level in normal discs (Fig. 8b–b′′). Next, we examined the effects of Diap1 overexpression in *Ciao1*^*Δ60*^ mutant clones by utilizing the MARCM mosaic analysis technique^34^. Control *Ciao1*^*+*^ clones showed normal CycE pattern, and *Ciao1* mutant clones generated by MARCM method showed CycE reduction (Fig. 8c’, d’). However, there was elevated CycE level in *Ciao1*^*Δ60*^ clones expressing Diap1 (Fig. 8e′). Hence, Diap1 overexpression can induce CycE expression in the absence of Ciao1, which is consistent with the partial rescue of *Ciao1 RNAi* phenotype by Diap1 overexpression. These results suggest that Ciao1 may not be required for CycE expression and that the reduced CycE in *Ciao1* clones may be due to reduced Diap1.

## Discussion

Ciao1 is a part of the CIA machinery required for biogenesis of extra-mitochondrial Fe/S proteins such as XPD. Despite studies in the mammalian system, not much is known about the role of Ciao1 in organ growth. In our study, we demonstrate that Ciao1 is essential for regulation of cell survival and proliferation in developing *Drosophila* organs.

Our results show that Ciao1 knockdown in the differentiating eye by *GMR-Gal4* has little effect in adult eye morphology. However, in undifferentiated cells, Ciao1 reduction leads to reduced organ size. Such outcomes suggest that Ciao1 is mainly required for survival and proliferation of undifferentiated cells. The preferential role of Ciao1 for cell survival and proliferation is consistent with the result that Ciao1 loss-of-function leads to developmental defects with reduced Diap1 and CycE levels.

Loss of Ciao1 or Xpd leads to similar defects in organ growth, consistent with their genetic interaction. Ciao1 and Xpd may function together as a protein complex or in sequence. Developmental defects of p52 TFIIH subunit mutant are known to be suppressed by overexpression of another subunit p8/TTDA, suggesting that loss of a subunit function can be complemented by providing another factor of the complex^35^. Suppression of *Xpd RNAi* phenotype by Ciao1 overexpression and vice versa provides additional evidence for such functional relationship. Since the eye phenotype of Crb^intra^ overexpression is partially suppressed by Ciao1, Crb^intra^ phenotype might be mediated, in part, through Ciao1. Crb^intra^ overexpression results in overproliferation in the eye disc by inhibiting Hippo signaling, although adult eyes are not enlarged due to massive retinal disruption during pupal development. Since *Ciao1 RNAi* causes reduction of Diap1 and CycE levels, suppression of Crb^intra^ eye phenotypes by *Ciao1 RNAi* may be due to increased apoptosis in overproliferating cells and reduction of CycE, thus resulting in proper cell numbers in the eye discs. Xpd overexpression can also restore *Ciao1 RNAi* phenotype (Fig. 4g–g′), indicating that high level of Xpd can bypass Ciao1 reduction. Interestingly, Ciao1 overexpression can also suppress *Xpd RNAi* phenotype. These data suggest that high levels of Ciao1 and Xpd can mutually promote their function. In this scenario, overexpression of Xpd may promote the function of residual Ciao1 or bypass the Ciao1 reduction through an alternative pathway. Likewise, Ciao1 overexpression can compensate Xpd reduction. Mammalian CIA complex is required for integration of XPD into TFIIH in the cytoplasm, subsequently leading to its nuclear translocation^11,12^. In this model, CIA targeting complex is necessary for XPD function but not vice versa. However, our genetic data suggest a mutual interaction between Ciao1 and Xpd. It is an interesting question whether such feedback regulation exists between human Ciao1 and XPD. It is noteworthy that double knockdown of Ciao1 and Xpd does not show significant enhancement of *Ciao1* or *Xpd RNAi* alone (Fig. 4d). Hence, Ciao1 and Xpd appear to function in the same pathway rather than in two parallel pathways. Crb^intra^ phenotype is also suppressed by reducing Galla function. Furthermore, both Galla1 and Galla2 show similar rescue effects of Ciao1 reduction in vivo (Fig. S1b–f), suggesting that they have overlapping functions to compensate the loss of Ciao1.

In support of the roles of Ciao1 and Xpd in cell survival, loss of Ciao1 or Xpd shows increased apoptotic activity (Fig. 6b–c′′′). Clonal analysis of both *Ciao1* and *Xpd* mutants reveal similar reduction in Diap1 expression. However, Ciao1 and Xpd differ in the overexpression effect of Diap1 and p35 (Fig. 7e–j). Overexpression of p35 has little effect on both *Ciao1 RNAi* and *Xpd RNAi* eye phenotypes. In contrast to p35, Diap1 overexpression efficiently suppressed growth defects resulting from *Ciao1 RNAi* but not *Xpd RNAi* (Fig. 7e–f, j). Based on the apoptotic activity, it appears that both Ciao1 and Xpd show caspase-dependent apoptosis; however, their differences may lie in the insensitivity to p35 or Diap1. The results suggest that Xpd may regulate Diap1 along with an additional cell survival factor that may be unnecessary for Ciao1.

Our data for suppression of *Ciao1 RNAi* phenotype by either CycE or Diap1 overexpression raises a possibility that reduced CycE level in *Ciao1*^*Δ60*^ mutant clones might be a secondary effect of cell death. Indeed, Diap1 overexpression in the *Ciao1*^*Δ60*^ MARCM clones leads to increased CycE expression. These data suggest that reduced CycE level in *Ciao1*^*Δ60*^ clones might be a consequence of cell death from decreased Diap1. Since CycE can be strongly induced by Diap1 overexpression in the *Ciao1*^*Δ60*^ null mutant clones, Ciao1 seems to be dispensable for CycE expression. Despite severe reduction of the posterior compartment of wing discs in *en* > *Ciao1 RNAi*, the level of cell proliferation marked by PH3 staining is not significantly reduced (Fig. 2m–m′′). This result seems to be consistent with the observation that Ciao1 may not be crucial for cell proliferation as long as cells can survive normally. However, it is important to note that the induction of CycE in *Ciao1*^*Δ60*^ MARCM clones was achieved by Diap1 overexpression, which is a nonphysiological condition. On the other hand, *Ciao1*^*Δ60*^ clones show significant CycE reduction, and overexpression of CycE efficiently rescues Ciao1 reduction phenotype in the absence of Diap1 overexpression (Fig. 5a–a′′, g). Hence, we cannot exclude the possibility that Ciao1 may also be required for proper CycE maintenance. Taken together, we propose that Ciao1 is required for regulating Diap1 and CycE level for cell survival and proliferation, respectively. In this model, the primary function of Ciao1 in developing organs might be to regulate the Diap1 level, thus allowing cell survival for CycE expression to promote growth.

Crb is an upstream regulator of Hippo signaling that leads to inhibition of Yorkie-mediated transcriptional activation. Since both *cycE* and *diap1* are target genes for Yorkie^20–22^, Ciao1 and Xpd may function in the Hippo-Yorkie pathway. However, we could not detect significant changes in Hippo or Yorkie expression in *Ciao1* mutant clones. Further, *Ciao1 RNAi* eye phenotype was not enhanced by Warts overexpression. Thus, Ciao1 functions may not be directly associated with Hippo-Yorkie signaling. Instead, our work suggests that Crb may also be involved in regulation of cell proliferation and survival through a new mechanism by affecting Ciao1, Galla, and Xpd, although the role of Hippo signaling cannot be excluded.

Mammalian Ciao1, MIP18, and XPD form a TFIIH-independent MMXD complex. In this case, Ciao1 and Xpd might be independent of TFIIH-dependent transcription^12,13^. However, there is evidence that the CIA complex containing MMXD proteins is also important for the TFIIH function in transcription by modulating XPD^12^. It remains to be studied whether Ciao1 and Xpd participate in Diap1 expression by regulating TFIIH or in a posttranslational regulation of Diap1 protein level.

Control of organ growth is essential in development, and disruption of well-coordinated organ growth results in various critical diseases. Our study provides evidence for cell survival and growth control roles of Ciao1 which is thus far known mainly for its function in the CIA complex. It would be interesting to see whether XP syndromes are related to the interaction between Ciao1 and XPD and their regulation of CycE and IAP family apoptosis inhibitors.

## Materials and methods

### Fly stocks

All *Drosophila* strains were grown and maintained at 25 °C. Following fly stocks were used for the experiments: *Ciao1* RNAi (Vienna *Drosophila* Resource Center, v32020 and v105939), *Xpd* RNAi (Vienna *Drosophila* Resource Center v106998), *UAS-CycE* (BDSC 4781), *UAS-Diap1, UAS-p35, ey-Gal4*, *GMR-Gal4, en-Gal4, ptc-Gal4*, *nub-Gal4*, and *FRT42D M(2)53*^*1*^ (BDSC 5698) were obtained from the Bloomington stock center. For overexpression of Crb^intra^, *UAS-Crb*^*intra*^ was crossed with *GMR-Gal4* (Bloomington). *Xpd*^*p*^ flies were a kind donation from Dr. Beat Suter. To construct the transgenic lines, *UAS-Ciao1* and *UAS-Xpd*, full length *Ciao1* and *Xpd* cDNA (from the *Drosophila Genome Research Center*) were cloned into pUAST vector.

### Generation of *Ciao1* null mutant

*Ciao1*^*△60*^ deletion allele was generated by imprecise excision of the P-element insertion *Ciao1*^*EY11076*^. Potential excision lines were identified by the loss of *w* markers, and genomic DNA from these lines was used as PCR templates. Deletion break points were confirmed by sequencing. Loss of Ciao1 protein was checked by immuostaining of *Ciao1* mutant clones in the eye disc. RT-PCR and western blot analysis were also performed with heterozygote flies.

### Clonal analysis

For generation of *Ciao1* mutant clones, *FRT42D Ciao1*^*△60*^*/CyO* was crossed with either *yw eyflp; FRT42D ubiGFP* or *hsflp; FRT42D ubiGFP*. For generation of *Xpd* mutant clones, *FRT42D Xpd*^*p*^*/CyO* was crossed with either *yw eyflp; FRT42D ubiGFP* or *hsflp; FRT42D ubiGFP*. For induction of mitotic recombination, first-instar larvae were treated with heat shock for 60 min at 37 °C for 3 consecutive days during first- and second-instar larval stages. For EGUF clones, *FRT42D GMR-hid l(2)CL-R*^*1*^*/CyO; ey-Gal4 UAS-flp* flies (BDSC 5251) were used to cross with *FRT42D Ciao1*^*△60*^*/CyO*. MARCM clones were generated by utilizing the following fly lines: (a) *yw hsflp, tub-Gal4, ubiGFP/FM7 GFP; FRT42D tubP-Gal80/CyO, (b) FRT42D Ciao1*^*△60*^*/CyO*, and (c) *FRT42D Ciao1*^*△60*^*/CyO; UAS-Diap1/TM6B*. The MARCM-ready flies (a) were crossed with (b) to generate *Ciao1*^*△60*^ clones. To express Diap1 in *Ciao1*^*△60*^ clones, flies (a) were crossed with (c). The clones were induced by heat-shock treatment for 60 min at 37 °C during the first-instar larval stage.

### Generation of the Ciao1 antibody

Whole coding sequence of Ciao1 was amplified and cloned into pMAL-c2 through BamHI and SalI sites. MBP-Ciao1 fusion protein was expressed in BL21 by IPTG induction, and the purified protein was used to immunize rats.

### Immunostaining

Third-instar larvae were dissected in ice-cold phosphate-buffered saline (PBS). Collected tissues were fixed at 4% paraformaldehyde in PBS for 20 min on ice. After washing twice with PBS, fixed tissues were blocked in 5% normal goat serum/PBT (PBS + 0.3% Triton-X100) for 2–4 h at 4 °C. Samples were incubated with primary antibodies in 5% NGS/PBT at 4 °C overnight. The following antibodies were used for staining: rat anti-Ciao1 (1:100), mouse anti-GFP (1:100) (ab1218, Abcam), sheep anti-GFP (1:100) (4745-1051, BioRad), mouse anti-Elav (1:50) (from K.O. Cho), rabbit anti-BarH1 (1:100) (from J.K. Kang), rabbit anti-PH3 (1:200) (06-570, Millipore), rabbit anti-CycE (1:100) (sc-33748, Santa Cruz), rabbit anti-Cleaved Dcp-1 (Asp216) (9578, Cell Signaling), and mouse anti-Diap1 (1:100) (from B. Hay). After washing three times with PBT, secondary antibodies conjugated with FITC (1:100), Cy3 (1:600), or Cy5 (1:500) (Alexa Fluor, Molecular Probes) were incubated for at least 2 h at room temperature. After washing four times with PBT, Vectashield with DAPI (H-1200, Vector Laboratories) was used to mount the prepared samples. Fluorescent images were acquired using Carl Zeiss LSM710 confocal microscope and ZEN software.

### Cell culture, transfection, immunoprecipitation, and western blot analysis

*Drosophila* S2 cells were cultured in M3 media (Sigma) with 10% Insect medium supplement (Sigma). Transfection was carried out with Effectene reagent (Qiagen) according to manufacturer’s instructions. Total of 1–2 μg DNA was used for each transfection. For immunoprecipitation, cells were lysed in 0.1% CHAPS buffer, and the lysates were precleared by incubating with protein G-sepharose beads (Roche) for 1 h at 4 °C. The G-sepharose beads were immunoprecipitated with anti-Myc (Abcam) or anti-Flag (Sigma) at 4 °C for 1 h. The immunoprecipitates captured by protein G-sepharose were incubated with the clear lysates overnight at 4 °C. Immunoprecipitates were washed three times in cold IP buffer. The samples were boiled in protein loading buffer at 94 °C for 5 min and then subjected to SDS-PAGE. Western blot was performed with mouse Flag (Sigma) or mouse V5 at 1:5000 (Invitrogen).

### In vitro GST pull-down assays

For GST pull-down, IPTG-inducible R2 cells (BL21 derivative) were transformed with plasmids for MBP-Ciao1, GST-Galla1-N, GST-Galla1-C, MBP-Xpd, and GST-Ciao1. Bacterial cell lysates were prepared as described^36^. Pull-down buffer at following condition was used: 20 mM Tris pH 7.5, 150 mM NaCl, 0.5 mM EDTA, 10% glycerol, 0.1% Triton-X100, 1 mM DTT, and protease inhibitor cocktail (Roche). For western blot analysis, rabbit anti-MBP antibody (1:10,000) (E8030S, NEB), mouse anti-GST antibody (1:5000) (Santa Cruz), and secondary anti-rabbit and anti-mouse antibody conjugated with HRP (1:10,000) (711-035-152 and 715-035-151, Jackson) were used.

### Imaging and statistical analysis

All fluorescent images were acquired using Carl Zeiss LSM710. Adult eye and wing samples were photographed at different levels ranging from top to bottom with the Axiocam software (Zeiss). Multilevel images were combined using the Zeren Stacker software. Quantification of the adult eye size was performed using the Image J software. Statistical analysis shown in Figs. 5i and 7j were evaluated using GraphPad Prism 8 (http://www.graphpad.com). Mean values of the data are presented with standard error of mean (±s.e.m.) where indicated. Organ size phenotype was quantified relative to the size of wild-type organs, in which the error bar of the control was calculated as standard error of mean (± s.e.m.) as indicated. Statistical significance was evaluated by unpaired one-tailed Student’s *t* test using Microsoft Office Excel and are indicated as ***p < 0.001, **p < 0.01 and *p < 0.05.

## Supplementary information

Supplementary Figure Legends

Figure S1

Figure S2

Figure S3

Figure S4

Figure S5
