# Supplementary material for: Ciao1 interacts with Crumbs and Xpd to regulate organ growth in Drosophila
Source: Cell Death Dis. 2020 May 13;11(5):365. doi: 10.1038/s41419-020-2564-3 (PMC7220951; doi:10.1038/s41419-020-2564-3)
Supplement: Supplementary file 1 — Supplementary Figure Legends [file 41419_2020_2564_MOESM1_ESM.docx]

**Supplementary Figure Legends**

**Supplementary Figure 1. Ciao1 interaction with Galla1 and rescue of *ey>Ciao1 RNAi* by Galla1 or Galla2 overexpression.**

(a) GST-pull down assay shows Ciao1 preferential binding to Galla1 compared with Galla2. (b) Small eye phenotype of *ey>Ciao1 RNAi*. (c) Rescue of *ey>Ciao1 RNAi* small eye by *UAS-galla1*. (d) *ey>galla1*. (e) Small eye of *ey>Ciao1 RNAi* is rescued by *UAS-galla2*. (f) *ey>galla2*. Scale bar, 200 µm (b-f).

**Supplementary Figure 2. Loss of Ciao1 has little effect on cell size.**

(a-b”) Armadillo (Arm) staining of *Ciao1* mutant clones. (a) Clones in the wing imaginal disc marked by loss of GFP. (a’) Arm staining. (a”) Merge. (b) Higher magnification view of clones marked in (a). (b’) Arm staining shows no significant difference in the cell size of mutant clones when compared to that of adjacent wild-type cells. (b”) Merge. Scale bar, 50 µm (a-a”). (c-e) Effects of *Ciao1* *RNAi* in cell density. The asterisk marked in (c) *ptc>GFP* and (d) *ptc>Ciao1 RNAi* indicate the sampled region between L3 and L4 selected for cell counting. High magnification view of sampled region showing cell density for (c’) *ptc>GFP* and (d’) *ptc>Ciao1 RNAi.* Scale bar, 100 µm (c-d). (e) Quantification of cell density in the sampled wing region. *ptc>+,* *ptc>GFP,* *ptc>Ciao1 RNAi,* n=12. All data represent the mean and standard error of mean (±s.e.m.), and p-values were calculated using the Student’s t-test. NS, not significant (p>0.05).

**Supplementary Figure 3. Ciao1 antibody testing and *Xpd RNAi* phenotypes in eye/wing.**

(a-a”) Ciao1 antibody was generated and tested for its specificity in tissues. *Ciao1^Δ60^* mutant clones (arrows), marked by GFP-negative regions (a), show strong reduction in the Ciao1 level (a’). (a”) Merge. Scale bar, 20 µm (a-a”). (b-e) *Xpd RNAi* phenotypes in eye and wing. (b) *ey>+* control eye. (c) Small eye phenotype of *ey>Xpd RNAi.* Scale bar, 200 µm (b-c). (d) *nub>Xpd RNAi* flies exhibit defective small wings. (e) Progenies of *Xpd RNAi* flies crossed with *MS1096-Gal4* driver also show abnormal smaller wings.

**Supplementary Figure 4. Control clones show no change in staining of CycE and Diap1.**

(a-b”) Control wild-type clones show no change in the CycE level. (a) Clones marked by loss of GFP. (a’) No changes in CycE staining of control clones. (a”) Merge. (b-b”) Control wild-type clones show no changes in the Diap1 level. (b) Clones marked by loss of GFP. (b’) No changes in Diap1 staining of control clones. (b”) Merge. Scale bar, 50 µm (a-b”).

**Supplementary Figure 5. Effects of Ciao1 (or Xpd) overexpression on CycE level under *Xpd RNAi (or Ciao1 RNAi)* background.**

Third instar larva eye discs were stained by DAPI and anti-CycE antibody. (a) *ey>+* control shows enhanced CycE expression along the second mitotic wave (arrow). (b) *ey>Xpd RNAi* eye disc has reduced size with abnormal disc shape. Very weak or no second mitotic wave with CycE can be detected. (c) *ey>Xpd RNAi, Ciao1* eye disc shows rescue of the disc size and CycE staining along the second mitotic wave (arrow). (d) *ey>Ciao1 RNAi* eye disc shows abnormal shape and size reduction. The second mitotic wave with high CycE levels is not clearly detected. (e) *ey>Ciao1 RNAi, Xpd* eye disc shows recovery of the disc shape and size. High levels of CycE are detected in the second mitotic wave (arrow). Scale bar, 50 µm (a-e).
