# Supplementary figures and images for: Ciao1 interacts with Crumbs and Xpd to regulate organ growth in Drosophila
Source: Cell Death Dis. 2020 May 13;11(5):365. doi: 10.1038/s41419-020-2564-3 (PMC7220951; doi:10.1038/s41419-020-2564-3)

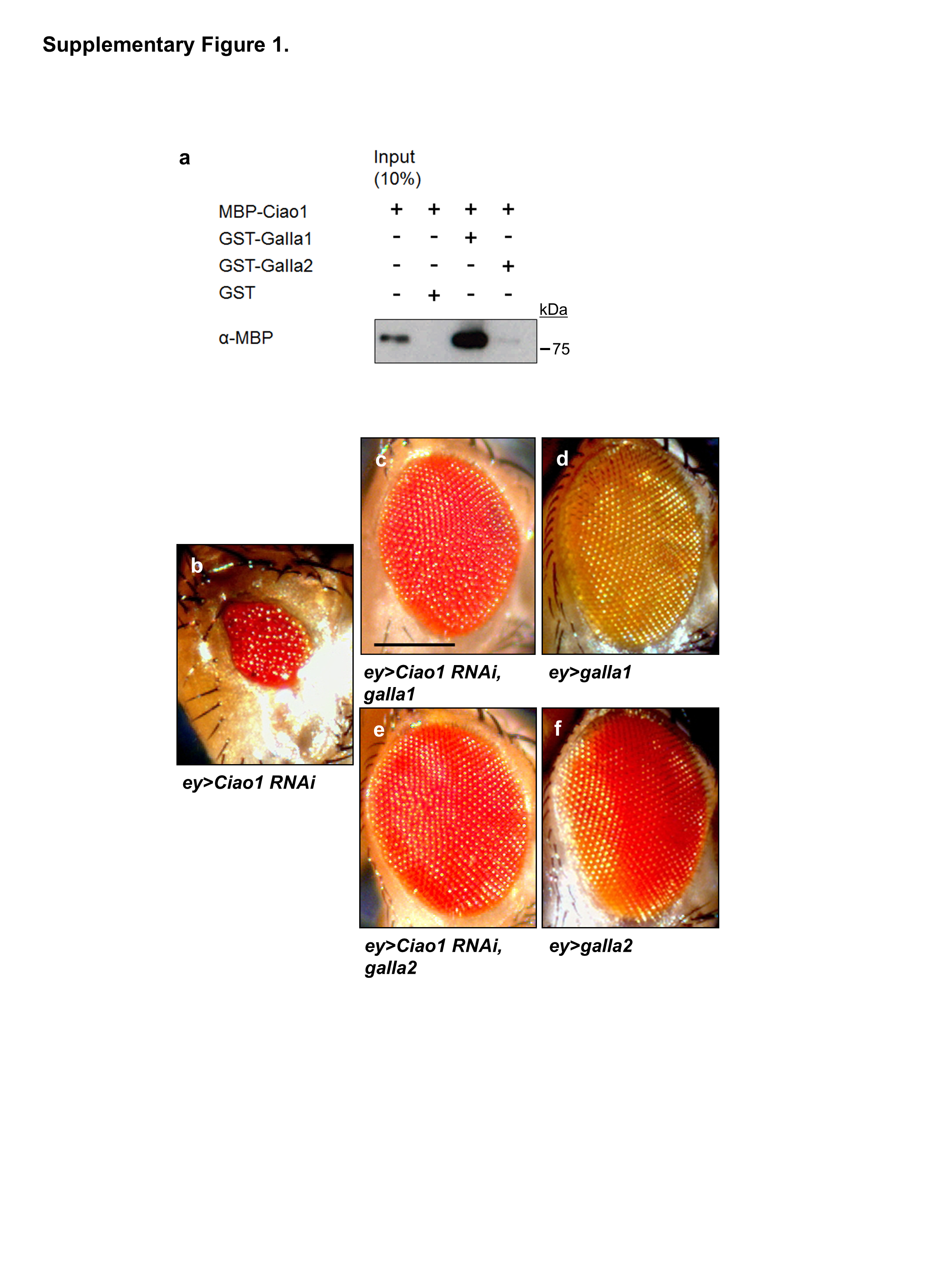

Supplement: Supplementary file 2 — Figure S1 [file 41419_2020_2564_MOESM2_ESM.tif]

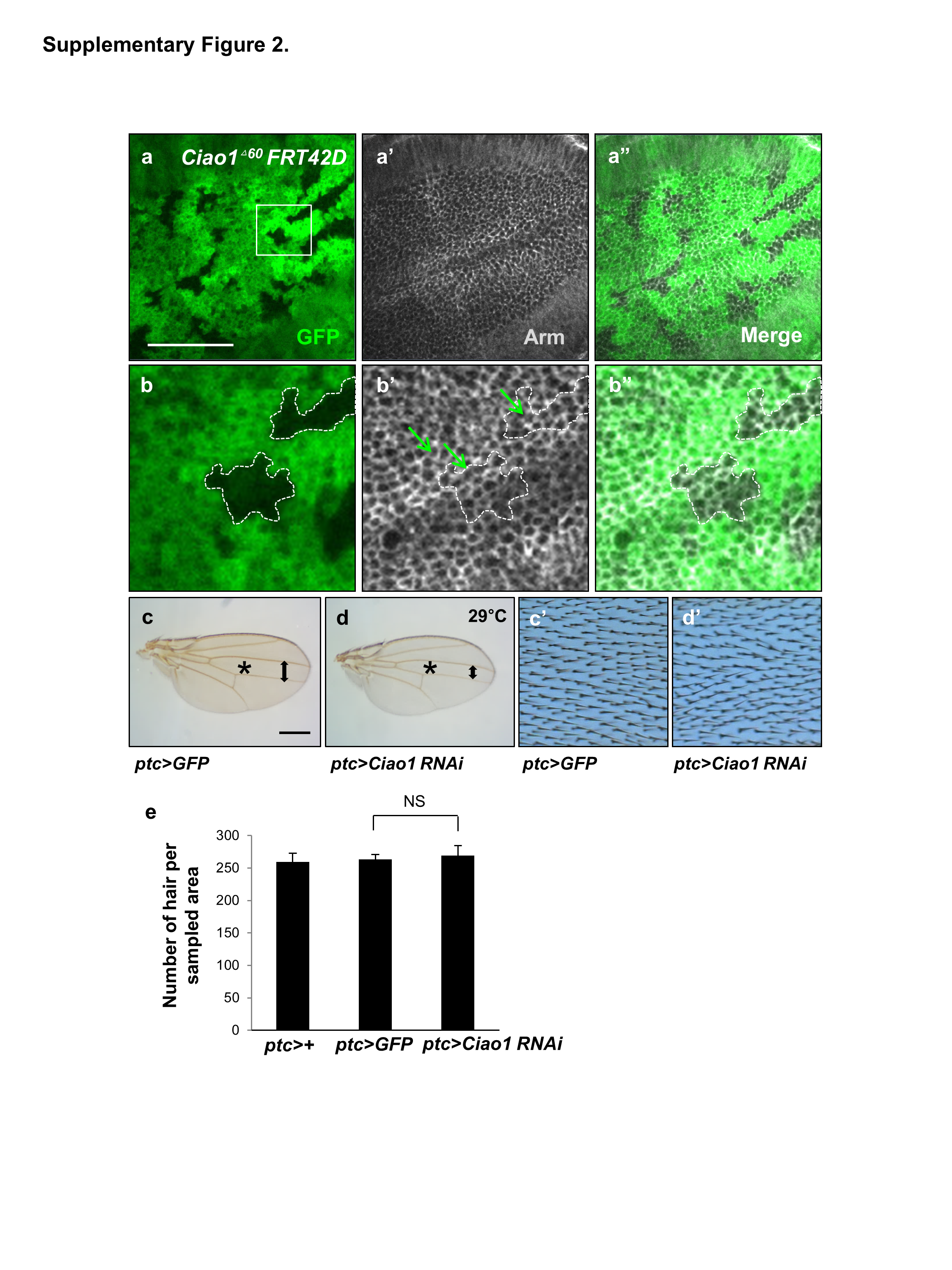

Supplement: Supplementary file 3 — Figure S2 [file 41419_2020_2564_MOESM3_ESM.tif]

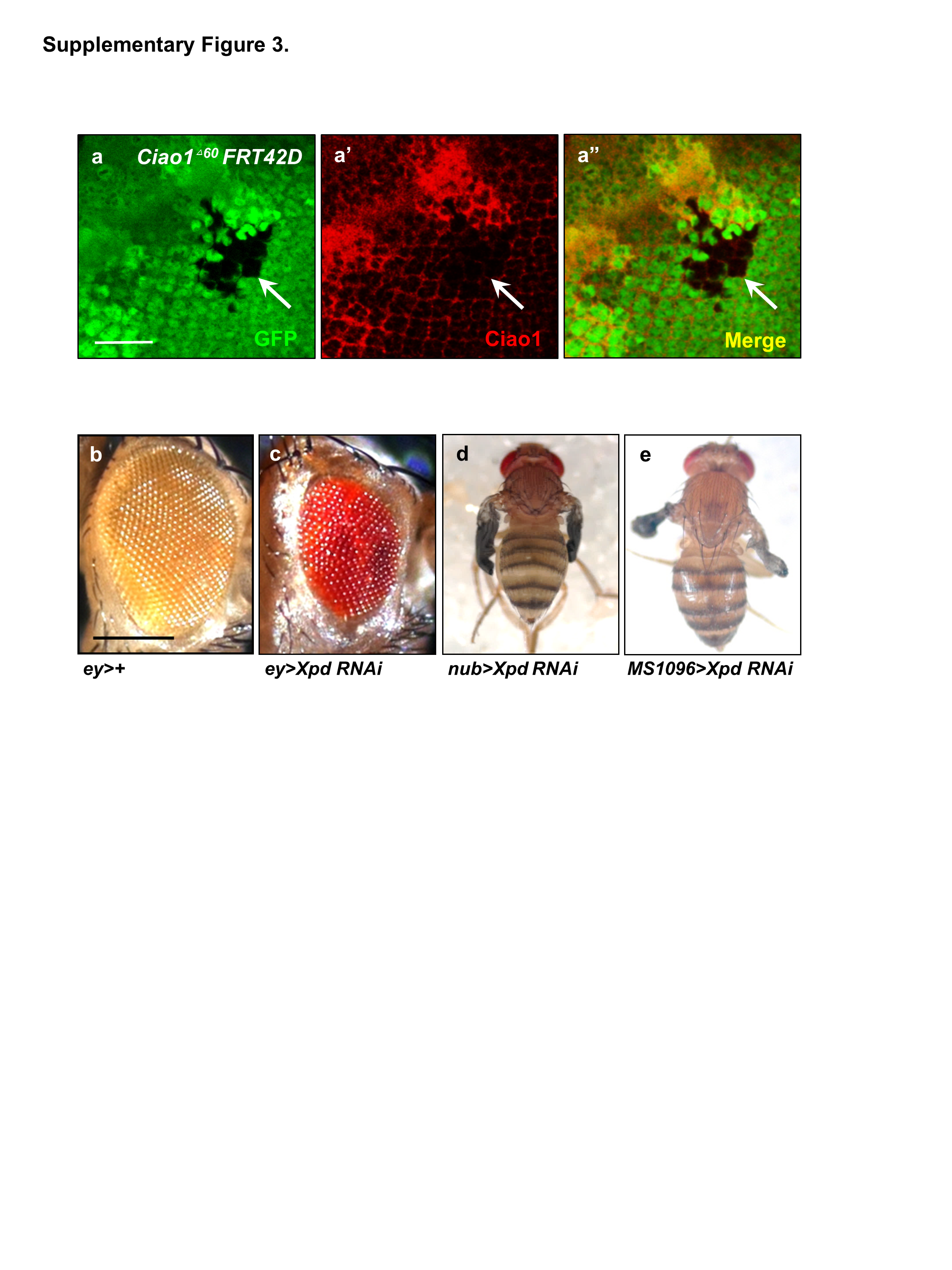

Supplement: Supplementary file 4 — Figure S3 [file 41419_2020_2564_MOESM4_ESM.tif]

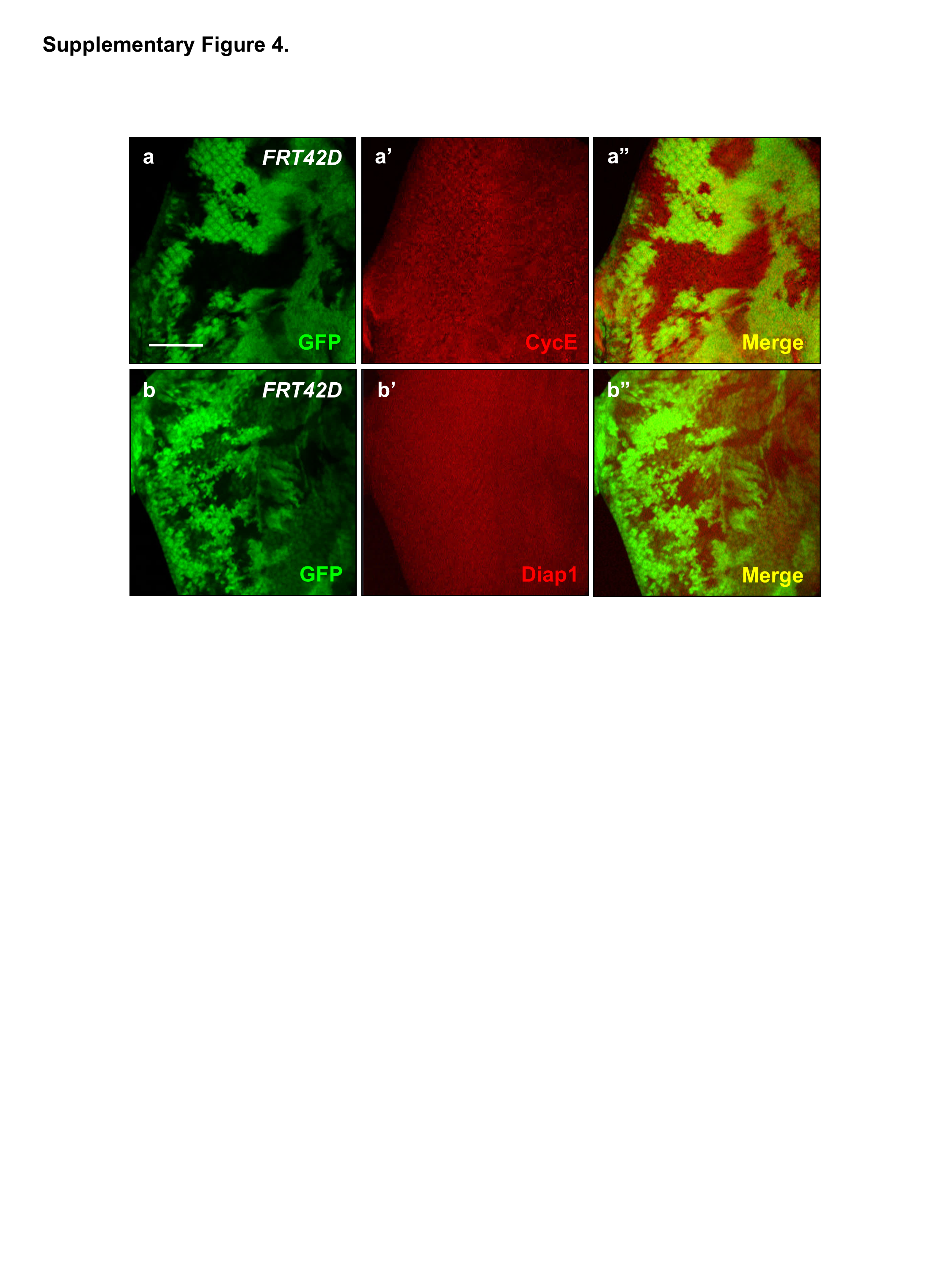

Supplement: Supplementary file 5 — Figure S4 [file 41419_2020_2564_MOESM5_ESM.tif]

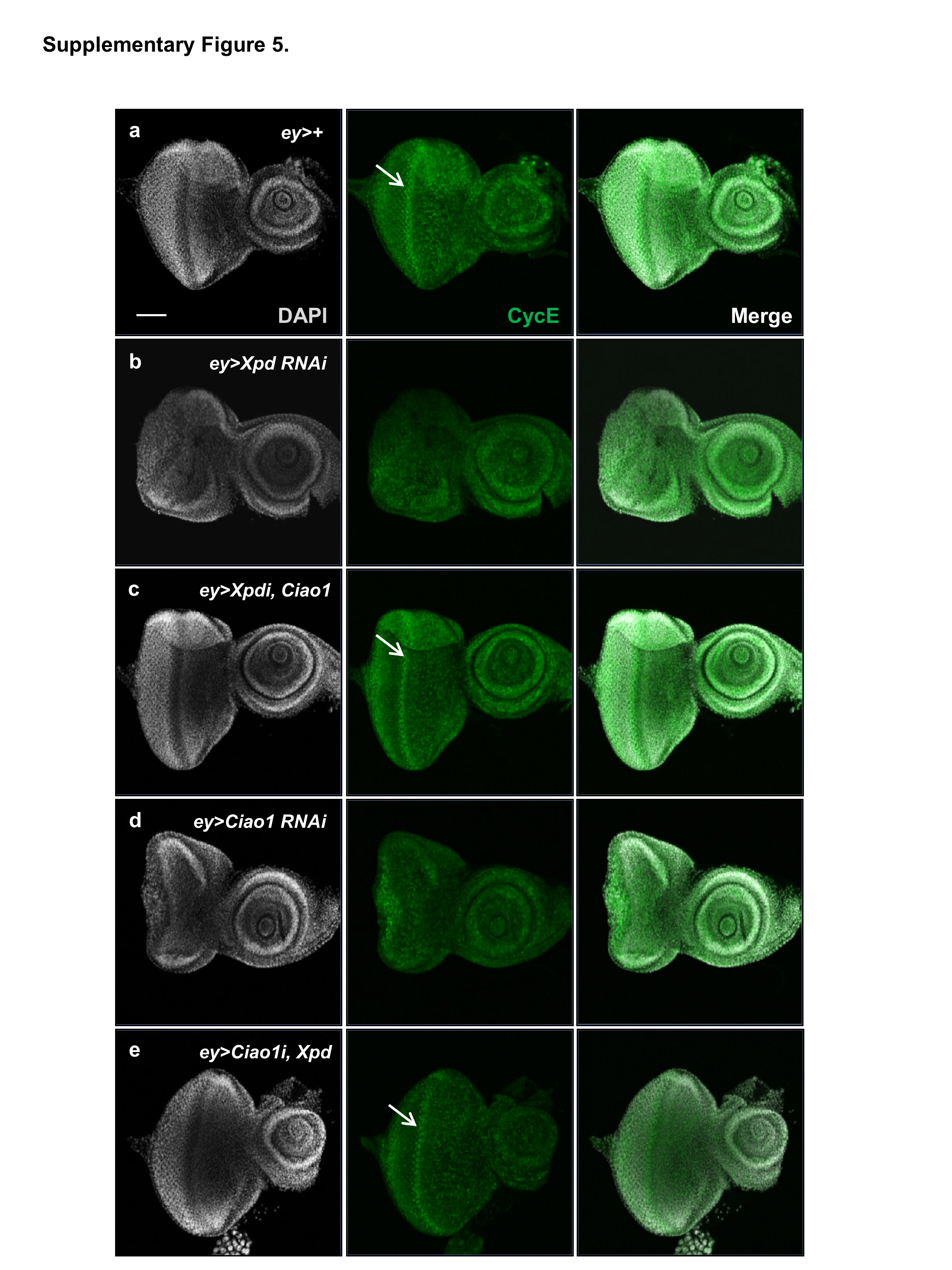

Supplement: Supplementary file 6 — Figure S5 [file 41419_2020_2564_MOESM6_ESM.tif]
